# Cannabinoid Regulation of Fear and Anxiety: an Update

**DOI:** 10.1007/s11920-019-1026-z

**Published:** 2019-04-27

**Authors:** Eleni P. Papagianni, Carl W. Stevenson

**Affiliations:** School of Biosciences, University of Nottingham, Sutton Bonington Campus, Loughborough, LE12 5RD UK

**Keywords:** Cannabidiol, Consolidation, Endocannabinoid, Extinction, Fear conditioning, Reconsolidation

## Abstract

**Purpose of Review:**

Anxiety- and trauma-related disorders are prevalent and debilitating mental illnesses associated with a significant socioeconomic burden. Current treatment approaches often have inadequate therapeutic responses, leading to symptom relapse. Here we review recent preclinical and clinical findings on the potential of cannabinoids as novel therapeutics for regulating fear and anxiety.

**Recent Findings:**

Evidence from preclinical studies has shown that the non-psychotropic phytocannabinoid cannabidiol and the endocannabinoid anandamide have acute anxiolytic effects and also regulate learned fear by dampening its expression, enhancing its extinction and disrupting its reconsolidation. The findings from the relevant clinical literature are still very preliminary but are nonetheless encouraging.

**Summary:**

Based on this preclinical evidence, larger-scale placebo-controlled clinical studies are warranted to investigate the effects of cannabidiol in particular as an adjunct to psychological therapy or medication to determine its potential utility for treating anxiety-related disorders in the future.

## Introduction

Anxiety- and trauma-related disorders are the most common psychiatric diseases and are associated with inadequate treatment options and thus high social and economic costs. Psychological treatments are often limited or temporary in their effectiveness, while medications can lack efficacy or have unwanted side effects in a considerable number of patients. Psychological therapies can also be combined with medications to enhance treatment synergistically, but some medications can interfere with these therapies. Better options are therefore urgently needed for treating these disorders [1].

With the decriminalization of cannabis, availability of cannabis-derived chemicals (i.e. cannabinoids), and anecdotal evidence for the anxiolytic potential of cannabinoids all becoming ever more widespread, it is important to take stock of the empirical evidence to determine if cannabinoids can live up to their hype as an option for treating anxiety-related disorders in the future. In this narrative review, we begin by describing these disorders and the current therapeutic approaches used in their treatment. We then review the preclinical and clinical studies that have investigated cannabinoid regulation of fear and anxiety. We conclude by outlining future directions for driving forward this promising avenue of research.

## Anxiety- and Trauma-Related Disorders and Their Treatment: Current Therapeutic Approaches

Anxiety and fear are emotional responses that occur in anticipation of potential threat or when facing imminent danger, respectively. These responses are adaptive when they occur appropriately in response to relevant aversive stimuli, but they become maladaptive when expressed inappropriately under benign conditions and can lead to the development of anxiety- and trauma-related disorders [2]. The anxiety disorders include generalized anxiety, panic, social anxiety, phobias and separation anxiety, with post-traumatic stress-disorder (PTSD) and obsessive-compulsive disorder being related to but now classed separately from anxiety disorders. Collectively, these anxiety-related disorders are the most prevalent psychiatric diseases and are therefore a significant socioeconomic burden, given their high costs to the health care system and their association with long-term disability, lost work productivity and disrupted social relationships [3]. These disorders are associated with perturbed cognition and emotional regulation. For example, they share common psychological (e.g. excessive fear, apprehension, disturbed concentration and sleep) and somatic (e.g. tachycardia, heart palpitations, sweating) symptoms, with arousal and avoidance behaviour thought to predict long-term disability [4•, 5]. Symptom overlap among the different anxiety-related disorders and with other psychiatric diseases is a diagnostic challenge, while self-medication with alcohol and/or other drugs can progress to substance abuse and lead to significant co-morbidity between these diseases [4•, 6].

Anxiety-related disorders are treated using psychological therapies or/and medications. The various psychological approaches include cognitive behavioural therapy, exposure therapy, cognitive processing therapy and eye desensitization reprocessing, with the aim of reducing avoidance behaviour and distress [4•, 7]. Selective serotonin reuptake inhibitors (SSRIs) are typically the first choice of medication, but other types of anti-depressants can be used if the response to SSRI treatment is inadequate; selective noradrenaline reuptake inhibitors (SNRIs) are favoured over tricyclics and monoamine oxidase inhibitors due to their more favourable safety and tolerability profile. Other drug therapies include anti-seizure medications, serotonin1A (5-HT1A) receptor agonists (e.g. buspirone), short-term benzodiazepine treatment for acute anxiety and beta-blockers for reducing somatic symptoms [8, 9].

While psychological and pharmacological therapies are effective [7, 8], both treatment approaches have their drawbacks. The effects of certain psychological treatments (e.g. exposure therapy) can be short-lived, limited outside of the therapeutic context and hindered by drugs of abuse and even certain anxiolytics, all of which can result in symptom relapse after treatment [1]. Medications can lack or have incomplete therapeutic effects, which often take weeks to commence in the case of first-line SSRI or SNRI treatment. Moreover, these treatments can also cause adverse effects (e.g. anxiogenesis, insomnia, agitation, headache, appetite and gastrointestinal disturbances, sexual dysfunction) prior to the onset of or along with their therapeutic effects. Benzodiazepines can cause unwanted central nervous system depressant effects, tolerance and withdrawal with abrupt discontinuation and have abuse liability. This has limited their recent use to managing acute anxiety in the short-term until the onset of therapeutic effects with first-line SSRI/SNRI treatment [9]. Benzodiazepines may also enhance the risk of developing PTSD and co-morbid substance abuse disorders, worsen PTSD symptoms and reduce the efficacy of psychological therapies for PTSD treatment [10]. Taken together, these issues highlight the limitations of psychological therapies and medications currently used for treating anxiety-related disorders.

## Cannabinoids: a Brief Overview

*Cannabis sativa* is one of the oldest plants known for its recreational and purported medicinal properties. It consists of more than 400 chemicals known collectively as phytocannabinoids, over 100 of which are pharmacologically active. The psychoactive delta-9-tetrahydrocannabinol (THC) and the non-psychoactive cannabidiol (CBD) are the most abundant phytocannabinoids and are present in different ratios depending on the plant strain. Other phytocannabinoids that have been less well studied to date include tetrahydrocannabivarin, cannabigerol, cannabichromene and cannabicyclol. The isolation of phytocannabinoids led to the identification of the biological targets by which they exert their effects, including the cannabinoid type 1 (CB1) and type 2 (CB2) receptors. The discovery of endogenous ligands for these receptors, lipid messengers known as endocannabinoids, followed, and the best studied of these to date have been anandamide and 2-arachidonoylglycerol (2-AG) [11•].

Cannabinoids have attracted considerable interest as candidate therapeutics for a range of neurological and psychiatric disorders due to the ubiquitous nature of endocannabinoid signalling and CB1 receptor expression throughout the brain [12, 13]. CB1 (and CB2) receptors and the other molecular mediators underlying endocannabinoid signalling are expressed in brain areas important for cognition, emotional regulation, defensive behaviours and their accompanying physiological responses (e.g. prefrontal cortex, hippocampus, amygdala, bed nucleus of stria terminalis, striatum, hypothalamus, periaqueductal grey, midbrain serotonergic and adrenergic nuclei), while both phytocannabinoids and endocannabinoids also act at various non-cannabinoid targets expressed in these areas (see below). Thus, cannabinoids are well placed to modulate the aberrant neural circuit dynamics that have been implicated in anxiety-related disorders [2, 11•, 14].

## Phytocannabinoid Regulation of Fear and Anxiety: the Case for Cannabidiol

Although recreational cannabis use is rife worldwide, it can be associated with anxiety symptoms acutely [15]. In terms of the mechanism underlying this effect of cannabis, studies in healthy volunteers dating back several decades showed that THC and CBD have opposing effects on anxiety. THC is anxiogenic, but this effect is diminished when it is co-administered with CBD [16]. In contrast, CBD given alone has anxiolytic properties, particularly under circumstances or in response to stimuli which normally provoke anxiety. Both the anxiogenic and psychotropic effects of THC would appear to preclude its use for treating anxiety-related disorders, at least when administered on its own. However, the reported anxiolysis caused by CBD gave rise to a number of preclinical studies that investigated its effects in different rodent models of innate fear and anxiety-like behaviour (e.g. elevated plus maze, open field, light-dark test, predator exposure). The findings of these studies broadly confirmed the anxiolytic potential of CBD when given systemically or infused locally into various brain areas governing fear and anxiety [14]. Neuroimaging studies have shown that the anxiety-reducing effects of CBD are accompanied by altered blood flow to some of the homologous areas in humans [17–19]. CBD is devoid of abuse potential given its lack of rewarding effects [20–22]. It also has a favourable safety profile and was recently approved for the treatment of rare childhood seizure disorders [23, 24]. This makes CBD an attractive candidate therapeutic for treating anxiety-related disorders.

Studies using preclinical models of relevance to anxiety-related disorders characterized by abnormally strong and persistent fear memory (i.e. phobias, PTSD) have shown that CBD also regulates learned fear and its inhibition in different ways. During fear conditioning, a cue or context is paired with a noxious stimulus, resulting in the consolidation of an associative fear memory. Later cue presentation or context re-exposure alone initially results in conditioned fear responding and can also destabilize the memory trace, requiring its reconsolidation to maintain or update the fear memory. Repeatedly presenting the cue or prolonged context re-exposure also reduces fear responding through an inhibitory learning process known as extinction, which competes with the original memory to suppress fear responding and also forms the theoretical basis of exposure therapy. Reducing conditioned fear responding, disrupting reconsolidation and enhancing extinction are all potential strategies for acute or lasting symptom reduction in phobias and PTSD [1, 25].

Acute systemic CBD treatment or infusion of CBD into discrete areas of the fear circuit before or after conditioning reduces fear memory encoding [26–29], although the clinical relevance of interfering with the formation of fear memory is somewhat limited. CBD also reduces learned fear expression acutely when given systemically [30–33] or centrally into some [31, 34–36], but not all [31, 37], areas of the fear circuit. Reconsolidation is disrupted by CBD treatment after memory retrieval [38–40], while extinction is potentiated by CBD given systemically or centrally [33, 41–43], although these opposing effects of CBD both lead to reduced learned fear.

Given the wealth of preclinical evidence for the anxiolytic potential of CBD, it is perhaps not surprising that case reports and small-scale studies examining its effects in a range of anxiety-related disorders have recently emerged. Overall, their findings have indicated that CBD treatment provides symptom relief in these disorders [44–48]. However, it should be stressed that large-scale placebo-controlled studies are needed to confirm these preliminary, albeit encouraging, results.

A number of pharmacological mechanisms underpin the potential therapeutic effects of CBD generally [49], but its regulation of anxiety-like behaviour and learned fear processing involves 5-HT1A receptors, transient receptor potential vanilloid 1 (TRPV1) channels and endocannabinoid signalling. The acute effects of CBD given systemically on anxiety and learned fear expression have been shown to be dose-dependent, such that low and intermediate, but not high, doses are effective. These effects of low and intermediate doses of CBD are blocked by 5-HT1A receptor antagonists given systemically or locally into various relevant brain areas, whereas blocking TRPV1 receptors centrally allows for high doses of CBD to be effective. These results indicate that the anxiolytic effects of lower doses of CBD involve 5-HT1A receptor activation, whereas higher doses of CBD might not affect anxiety by also activating TRPV1 channels [14, 50].

In contrast to the acute anxiolytic effects of CBD, its enhancement of extinction and disruption of fear memory consolidation and reconsolidation involve cannabinoid receptors. CBD-induced disruption of consolidation is blocked by CB1 and CB2 receptor antagonists infused centrally [28]. Disruption of reconsolidation by CBD is also blocked by systemic or central CB1 receptor antagonist treatment [38, 51]. Extinction enhancement by CBD is blocked by central CB1 receptor antagonism [41, 42]. These results indicate that CBD regulation of learned fear processing is mediated at least in part by cannabinoid receptor activation. However, CBD shows little affinity for CB1 or CB2 receptors [52]. This suggests that its cannabinoid receptor-dependent effects on extinction and fear memory consolidation and reconsolidation occur indirectly by modulating endocannabinoid signalling, which we summarize below.

## Endocannabinoid Signalling: a Target for Regulating Fear and Anxiety

As alluded to above, endocannabinoid signalling involves endocannabinoid activation of cannabinoid receptors and other non-cannabinoid targets. 2-AG and anandamide are the best characterized endocannabinoids, and they have differing affinities for these targets. 2-AG acts as a full agonist at CB1 and CB2 receptors, while anandamide has lower affinity for cannabinoid receptors but acts as a full agonist at TRPV1 receptors [11•]. Endocannabinoid signalling differs from that of classical neurotransmitters in that they are synthesized on demand in post-synaptic neurons in response to neuronal activation and act on their targets located presynaptically or in the post-synaptic neuron itself to mediate retrograde or non-retrograde signalling, respectively. During retrograde signalling, endocannabinoids act on presynaptic CB1 receptors to suppress neurotransmitter release from excitatory (i.e. glutamatergic) or inhibitory (i.e. GABAergic) neurons. This retrograde signalling is involved in different forms of short-term (i.e. depolarization-induced suppression of excitation or inhibition) and long-term (i.e. homosynaptic glutamatergic or heterosynaptic GABAergic long-term depression) synaptic plasticity. During non-retrograde signalling, endocannabinoids act on post-synaptic cannabinoid receptors or TRPV1 channels. This non-retrograde signalling regulates self-inhibition via a CB1 and CB2 receptor-dependent reduction in excitability and also synaptic plasticity through a TRPV1-mediated form of long-term depression [53]. Endocannabinoid signalling is tightly regulated by transporters that remove endocannabinoids from the synapse and degradative enzymes that metabolize them. Monoacylglycerol lipase (MAGL) is found presynaptically and is the main enzyme responsible for metabolizing 2-AG, whereas fatty acid amide hydrolase (FAAH) is located post-synaptically and is the main enzyme that mediates anandamide degradation [54]. Other pathways are also involved in metabolizing endocannabinoids, with cyclooxygenase-2 (COX-2) degradation of anandamide and 2-AG [55, 56] recently implicated in regulating fear and anxiety (see below).

Endocannabinoid signalling is thus ideally positioned to modulate neuronal activity and synaptic plasticity in the fear and anxiety circuitry. Moreover, various gene variants associated with endocannabinoid transmission (e.g. FAAH, CB1 receptor) have been linked to anxiety-related disorders [57–60, 61••, 62]. PTSD has also been associated with decreased 2-AG levels in the circulation, while anandamide levels were related to certain PTSD symptoms [63, 64]. However, other evidence has shown increased endocannabinoid levels in PTSD [65]. Nevertheless, pharmacological manipulation of endocannabinoid signalling at the level of cannabinoid receptors, transporters and degradative enzymes is a potential strategy for regulating fear and anxiety.

In terms of the cannabinoid receptor-dependent effects of CBD on learned fear regulation described above, CBD increases anandamide levels by inhibiting its transporter-mediated reuptake and degradation by FAAH [66]. CBD also binds to the fatty acid binding proteins that transport anandamide intracellularly to FAAH for its degradation, which may play a role in the inhibition of anandamide metabolism by CBD. There is also evidence that CBD reduces MAGL-mediated degradation of 2-AG [67, 68]. However, whether these putative mechanisms are involved in CBD regulation of learned fear processing remains to be confirmed.

CB1 receptor agonists can have both anxiolytic and anxiogenic effects, depending on the dose, route of administration, differences in CB1 receptor sensitivity in different brain areas and the aversive nature of the behavioural testing paradigm used [69]. As is the case with CBD, anandamide has been shown to be anxiolytic at lower doses and anxiogenic at higher doses, with the former effect involving CB1 receptor activation and the latter effect involving TRPV1 channel activation [70]. This indicates that maintaining the balance between CB1 receptor and TRPV1 channel activation is crucial for regulating anxiety, given their opposing anxiolytic and anxiogenic effects [71–73].

Elevating anandamide levels systemically or centrally via the pharmacological inhibition of FAAH is well known to produce anxiolysis, particularly under more aversive conditions [74]. In contrast, the effects of inhibiting MAGL to potentiate 2-AG levels have not been as well characterized and the results to date have been less clear. Most studies have shown that increasing 2-AG levels by inhibiting MAGL has anxiolytic effects but some have shown no or even anxiogenic effects of MAGL inhibition [74–77]. Interestingly, a recent study showed anxiolytic effects of FAAH or MAGL inhibition but not with a dual FAAH/MAGL inhibitor [78]. COX-2 inhibition, which is better known for its anti-inflammatory effects by interfering with prostaglandin synthesis, is also associated with endocannabinoid-dependent anxiolysis. This has been demonstrated using substrate-selective COX-2 inhibitors that prevent the degradation of endocannabinoids without affecting prostaglandin synthesis [79]. However, other evidence indicates that the anxiolytic effect of a different substrate-specific COX-2 inhibitor occurred in an endocannabinoid-independent manner [80].

In terms of endocannabinoid regulation of learned fear processing, a seminal study by Marsicano et al. (2003) provided compelling evidence that endocannabinoid signalling via CB1 receptors is crucial for fear extinction. CB1 receptor-deficient mice, or wild-type controls given a CB1 receptor antagonist, showed impaired fear extinction. Endocannabinoid levels were found to be elevated by extinction and also played a crucial role in modulating synaptic plasticity in the fear circuit in a CB1 receptor-dependent manner [81]. Subsequent studies have added to these findings by showing that genetic variants of FAAH resulting in elevated anandamide levels also enhance fear extinction [59, 82•]. Moreover, pharmacological FAAH inhibitors were found to enhance fear extinction in a CB1 receptor-dependent manner [41, 83–89], although the involvement of CB2 receptors in mediating anandamide regulation of fear extinction has not been characterized. In contrast to FAAH, genetic or pharmacological inhibition of MAGL impairs fear extinction [90, 91], suggesting opposing roles for anandamide and 2-AG in modulating fear extinction.

Endocannabinoid signalling has also been implicated in the consolidation and reconsolidation of fear memory. Inhibiting FAAH or MAGL to elevate anandamide or 2-AG levels was shown to enhance fear memory consolidation [92, 93], while FAAH inhibition also modulates the consolidation of stronger memory associated with fear generalization [28]. These effects likely involve both CB1 [86, 94–98] and CB2 [93, 99–101] receptor signalling. Fear memory reconsolidation is also modulated by endocannabinoid signalling as FAAH inhibition enhances the reconsolidation of fear memory [102]. However, the role of cannabinoid receptors in mediating this effect appears to be complex given that both agonists and antagonists have been shown to impair fear memory reconsolidation [85, 97, 102–104]. Post-retrieval fear memory destabilization, which is required to make reconsolidation of the fear memory trace amenable to pharmacological disruption, is enhanced by CB1 receptor activation [105, 106]. However, the involvement of MAGL/2-AG and CB2 receptor signalling in regulating the reconsolidation of fear memory remains to be elucidated.

## Conclusions

The evidence reviewed above demonstrates the potential utility of the phytocannabinoid cannabidiol and pharmacological inhibitors of FAAH, to elevate levels of the endocannabinoid anandamide, for the treatment of anxiety-related disorders in the future. Such cannabinoid-related medicines could be used in various ways to treat these disorders. Given their acute anxiolytic effects, cannabidiol and FAAH inhibitors could be used as adjuncts to first-line SSRI or SNRI treatment, which have a delayed therapeutic response. Such drugs could be an improvement over benzodiazepines, which have abuse liability, a less favourable side effect profile and can interfere with extinction, which forms the theoretical basis for exposure therapy used in the psychological treatment of certain anxiety-related disorders [1, 10]. In this respect, cannabinoids could be combined with existing or novel psychological therapies to facilitate extinction enhancement and/or fear memory reconsolidation disruption, both of which may result in a lasting reduction of fear. Cannabinoid-related medicines could also be given as anxiolytics on their own but further research is needed to determine their effects when given repeatedly as the few studies that have examined this issue have found mixed results [107–110]. The potential for certain adverse effects should also be examined more thoroughly given the reports of cannabinoid use being linked to executive dysfunction [111, 112]. Nevertheless, based on the evidence reviewed here, further research on cannabinoid regulation of fear and anxiety appears to be warranted and below we suggest various lines of enquiry for future work in this area.

The effects of these cannabinoids in other paradigms or measures of learned fear, and its return after extinction, should be characterized to determine if their reported anxiolytic effects are enduring and more widely applicable [28, 89, 113]. In terms of the pharmacological mechanisms underlying the indirect cannabinoid receptor-dependent effects of CBD on extinction and fear memory reconsolidation, further research is needed to determine which endocannabinoid or endocannabinoids are involved. The possibility that CB2 receptor signalling is involved in mediating the effects of CBD and anandamide should also be examined. This is because CB2 receptor activation might avoid the psychotropic effects associated with activating CB1 receptors [114], which also likely rules out the feasibility of using full CB1 receptor agonists for treating anxiety-related disorders. The synaptic plasticity mechanisms underlying the effects of cannabidiol and anandamide on extinction and fear memory reconsolidation, which might involve both cannabinoid receptor and TRPV1 signalling [115], also remain to be fully elucidated. Given the opposing roles of cannabinoid receptor and TRPV1 activation in regulating anxiety, novel drugs combining cannabinoid receptor agonist and TRPV1 antagonist properties may have synergistic anxiolytic effects [116]. Cannabinoids modulate the function of other neurotransmitters (e.g. 5-HT, noradrenaline, GABA) that underpin the therapeutic effects of currently available anxiolytic medications; therefore, understanding the relevant mechanisms involved may also lead to novel insights on the neurobiology of anxiety-related disorders [117, 118].

At the outset, we noted that the availability of phytocannabinoids and their use for self-medication in various anxiety-related disorders have recently become more commonplace. The anxiogenic effects linked to recreational cannabis use [15] likely involve a relatively high THC and low CBD content, but cannabis strains with different ratios of THC:CBD may have a more favourable anxiolytic profile. Interestingly, this could be investigated by systematically characterizing the effects of Sativex, a cannabis-derived extract with a ~ 1:1 ratio of THC and CBD that is already approved for clinical use to treat spasticity in multiple sclerosis, on anxiety and learned fear processing [119, 120]. “Pure” cannabidiol has also recently been approved for clinical use as Epidiolex for treating rare seizure disorders in children, which could facilitate its eventual use in treating anxiety-related disorders. Cannabis contains a plethora of other pharmacologically active phytocannabinoids that may also regulate fear and anxiety, but their effects have yet to be determined in relevant preclinical models.
